# Supplementary material for: Extracellular Self- and Non-Self DNA Involved in Damage Recognition in the Mistletoe Parasitism of Mesquite Trees
Source: Int J Mol Sci. 2023 Dec 29;25(1):457. doi: 10.3390/ijms25010457 (PMC10778891; doi:10.3390/ijms25010457)
Supplement: Supplementary file 1 [file ijms-25-00457-s001.zip › ijms-2742666-supplementary.pdf]

International Journal of *Molecular Sciences*

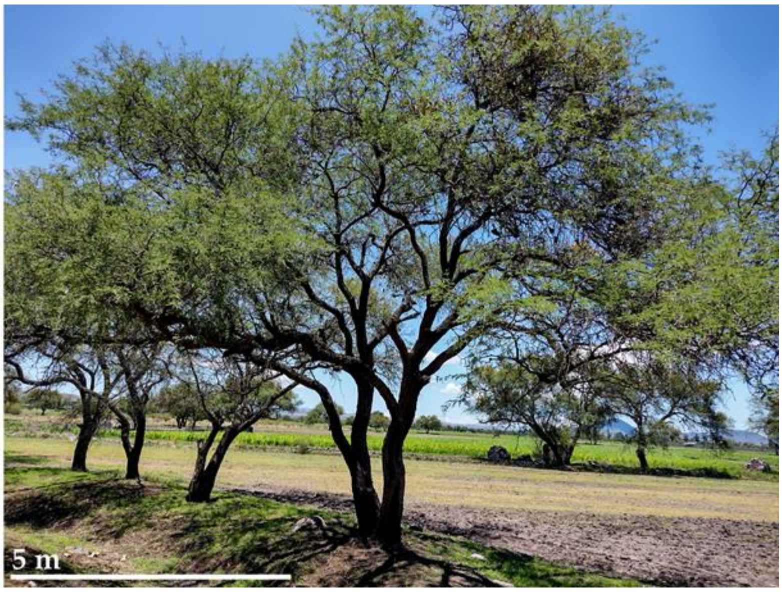

Figure S1. Mesquite trees used for the application of exDNA under field conditions.

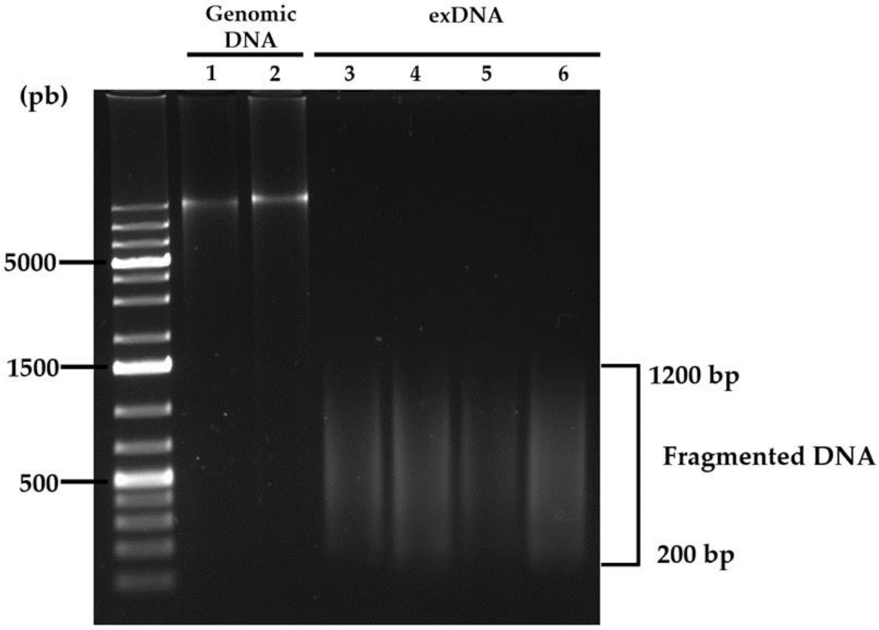

Figure S2. Agarose gel electrophoresis of self- and non-self exDNA. Genomic DNA was extracted from *P. laevigata* tree leaves (Line 1: 40 ng/ µL), and *P. calyculatus* mistletoe leaves (Line 2: 60 ng/ µL) that were fragmented by sonication to obtain the self- (Line 3, and 4: *P. laevigata*: 100 ng/µL or 200 ng/µL, respectively) and non-self exDNA (Line 5, and 6: *P. calyculatus*: 100 ng/µL or 200 ng/µL, respectively), that resulted in fragments around of 200-1200 bp. All samples were verified on 3.6% agarose gel with ethidium bromide staining.

Table S1. Primers sequences used for semi-quantitative RT-PCR analysis for *MAPK*s gene expression levels.

Name primer	Sequence (5′- 3′)	Protein	Amplicon size (bp)
*mapk*2 fw	agggttggccacttatggtg	Mitogen-activated protein kinase homolog MMK2-like	248 bp
*mpak*2 rv	catgatccatgtgccgaagc		
mapk4 fw	acggcggcaacagctacgataa	Serine/threonine-protein kinase 4 homolog A-like	469 bp

mapk4 rv	ctacgcaccacggttccactgac		
*mapk*10 fw	ctgatggatcgaaagccatt	Mitogen-activated protein kinase homolog D5 isoform X1, Protein homolog Mitogen-activated protein kinase 10	347 bp
*mapk*10 rv	gcatgttgctcaaagtcgaa		
*actin*1 fw	cctttatgctagtggccgca	Protein actin	261 bp
*actin*1 rv	tagcttttctcgacagctgaacta		
